# Differential Responses of *Cucurbita pepo* to *Podosphaera xanthii* Reveal the Mechanism of Powdery Mildew Disease Resistance in Pumpkin

**DOI:** 10.3389/fpls.2021.633221

**Published:** 2021-04-12

**Authors:** Shuwu Zhang, Jia Liu, Bingliang Xu, Jingjiang Zhou

**Affiliations:** ^1^College of Plant Protection, Gansu Agricultural University, Biocontrol Engineering Laboratory of Crop Diseases and Pests of Gansu Province, Lanzhou, China; ^2^Gansu Provincial Key Laboratory of Arid Land Crop Science, Gansu Agricultural University, Lanzhou, China

**Keywords:** *Cucurbita pepo*, powdery mildew, *Podosphaera xanthii*, disease incidence and index, reactive oxygen species, phenylpropanoids and SA pathways, antioxidative defense system, gene expression

## Abstract

Powdery mildew is one of the most destructive diseases and the major cause to the production losses of cucurbit worldwide. A number of strategies have been developed and applied to discover suitable and safer methods to manage the occurrence of powdery mildew disease in pumpkins (*Cucurbita pepo* L.), but information is limited in screening tolerant germplasms and exploring their mechanisms in preventing the disease occurrence at physiological, biochemical, and molecular levels. Therefore, we investigated the responses of two commercial pumpkin cultivars to *Podosphaera xanthii* infection. Compared with mock-inoculated seedlings, few small and sparse powdery areas were observed on the leaves of the Sixing F_1_ cultivar on the 13^th^ day after inoculation with *P. xanthii*, whereas a large number of diseased powdery areas and a layer of white powdery mildew were observed on the surface of Jin_12_ F_1_ leaves. The inoculation duration (7, 9, 11, and 13 days) significantly and continuously increased the disease incidence and index of pumpkin seedlings. The contents of H_2_O_2_, MDA, lignin, and total phenolics in the leaves of Sixing F_1_ and Jin_12_ F_1_ cultivars were markedly increased after inoculation with *P. xanthii*. However, the Sixing F_1_ cultivar exhibited much less reactive oxygen species (ROS) accumulation, a lower rate of lipid peroxidation, and a higher level of lignin and total phenolics contents after inoculation than the Jin_12_ F_1_ cultivar. Compared with untreated control pumpkin seedlings, significantly higher activities and gene expressions of the phenylpropanoids pathway enzymes (PAL and PPO), ROS scavenging defense enzymes (SOD, CAT, POD, and APX), and other salicylic acid (SA) signaling pathway marker genes were observed in the leaves of both cultivars after *P. xanthii* inoculation at different inoculation time points. These enhancements were significantly higher in Sixing F_1_ than Jin_12_ F_1_. Our results indicate that the Sixing F_1_ cultivar exhibited a much stronger ability in resistance to *P. xanthii* infection than the Jin_12_ F_1_ cultivar. Our results suggest that one possible mechanism of *C. pepo* cultivars to prevent the pathogen *P. xanthii* infection is by activating and enhancing the activity and gene expression of the phenylpropanoids pathway to synthesize phenolic substances and lignin, ROS scavenging defense enzymes to eliminate the harmful effects of ROS, and signaling pathway marker gene expression to improve plant disease resistance.

## Introduction

Powdery mildew is a common and widely distributed fungal disease that causes substantial yield and economic losses on a wide range of plants (Chen et al., 2020). Powdery mildew diseases are caused by many different species of fungi (Kusch and Panstruga, 2017; Babosha et al., 2020). *Podosphaera xanthii* has been considered as one of the most important pathogens that cause powdery mildew of cucurbit and reduce the cucurbit production worldwide (Tanaka et al., 2017). Pumpkin (*Cucurbita pepo* L.) is one of the most important vegetable crops for human nutrition worldwide (Hafez et al., 2018). Powdery mildew is one of the limiting factors that cause severely economic losses in pumpkin production by shortening the ripening and harvesting intervals, reducing photosynthesis and yields, and decreasing fruit quality in field and greenhouse (Cohen et al., 2000; Shi et al., 2007; Barickman et al., 2017). Normally, the pumpkin yield losses due to powdery mildew are 30–50% (El-Naggar et al., 2012).

In the past few years, strategies have been adapted to manage powdery mildew in agriculture, including the use of chemical and biological fungicides, and breeding resistance varieties (El-Alfy and Schlenk, 2002; Faostat, 2010). However, the application of chemical fungicides is not healthy and safe due to their hazardous effects on beneficial organisms, plants, animals, and humans, as well as pathogen resistance (Abdel-Monaim et al., 2012). The resistance to powdery mildew was first observed in cucumber (*Cucumis sativus* L. cv. Puerto Rico 37) (Smith, 1948); thereafter, a large number of resistant materials were found in South and East Asia (Morishita et al., 2003). Although biological control agents have been applied to control powdery mildew, their efficacy is low and affected by environmental conditions (Dik et al., 1998). Thus, effective and environmentally friendly control strategies are needed to overcome these problems. Screening of resistant pumpkin germplasm would be the best way for developing new cultivars to prevent the powdery mildew occurrence. But little is known about the specific knowledge for discovering and developing new resistant pumpkin varieties to prevent powdery mildew occurrence. The mechanisms and nature of pumpkin resistance to *P. xanthii* infection remain unresolved.

A number of studies have demonstrated that plants can develop appropriate defense mechanisms to recognize and resist against fungal infection through the activation of their complex defense responses (Dangl and Jones, 2001). One of the earliest responses is the rapid generation of reactive oxygen species (ROS) such as hydrogen peroxide (H_2_O_2_), hydroxyl radical (OH^–^), and superoxide anion (O^2–^) (Patykowski and Urbanek, 2003). ROS scavenging enzymes such as superoxide dismutase (SOD), peroxidase (POD), catalase (CAT), and ascorbate peroxidase (APX) play an essential role in regulating ROS levels and the extent of oxidative damage (Niu et al., 2018). The phenylpropanoid pathway is another defense response in higher plants (Irisarri et al., 2016; Hou et al., 2019). Phenylalanine ammonia-lyase (PAL) is the first enzyme that participates in the formation of a series of structurally different and defensive lignin and phenolic compounds (Vogt, 2010; Wang et al., 2013; Kamalipourazad et al., 2016; Han et al., 2017). Polyphenol oxidase (PPO) is another key enzyme in the synthesis of phenolic compounds for defending against pathogens in plants (Niu et al., 2018). In addition, previous studies revealed that SA plays an important role in fighting biotrophic pathogen infection and establishing systemic acquired resistance (SAR) (Dong, 1998; Edgar et al., 2006; Vlot et al., 2008; Fu et al., 2012). However, to our knowledge, there is little published information regarding the mechanisms of different cultivars of pumpkin *C. pepo* in resistance to *P. xanthii* infection through the phenylpropanoids and salicylic acid (SA) signaling pathways, and antioxidative defense systems.

Therefore, the aims of the present study were to (i) evaluate the ability and effectiveness of two commercial pumpkin cultivars in resistance to *P. xanthii* infection, (ii) determine the defense responses of the commercial pumpkin cultivars to the *P. xanthii* inoculation at different time points, and (iii) explore the possible mechanisms involved in two different pumpkin cultivars in response to *P. xanthii* infection at physiological, biochemical, and molecular levels.

## Materials and Methods

### Seeds Treatment

The seeds of two commercial pumpkin cultivars (Sixing F_1_ and Jin_12_ F_1_) were selected and kindly provided by Wuwei Golden Apple Co., Ltd. The seeds with a uniform size were surface-sterilized with 5% NaOCl (v/v) for 3 min. Thereafter, all the surface-sterilized seeds were rinsed with sterile water five times and soaked in sterile water for 12 h for germination.

### Greenhouse Experiments

The experiments were carried out in the greenhouse at Gansu Agricultural University in August 2013. The sterilized seeds were germinated in 9-cm Petri dishes and covered with two layers of absorbent cotton and blotter papers at a constant temperature of 25°C. The germinated seeds were planted in pots (12 cm in diameter) with 500 g of sterilized soil. Each pot was planted with 8 seeds and each cultivar had 12 pots (a total of 96 plants) after germination. The experiment was arranged in a completely randomized design in a greenhouse with the inside temperature maintained between 25 and 20°C (day and night), a photoperiod of 16L/8D, and a relative humidity of 60%. Irrigation was done twice weekly.

### *Podosphaera xanthii* Identification and Inoculum Preparation

Pumpkin leaves infected with powdery mildew were collected from the field (Wuwei, China) on July 15, 2013 for microscopic observations. The pathogen was identified to be *P. xanthii* according to the published papers (McGrath and Thomas, 1996; Shin, 2000). The artificial inoculation of pumpkin seedlings was performed manually by dusting the sporulated leaves, and the plants with the *P. xanthii* isolate were kept for 20 days in a greenhouse. Five plants at the four-leaf stage with relatively consistent growth were selected, and three leaves of each plant were inoculated with the suspension of powdery mildew fungal pathogen *P. xanthii* spores by the smear method. The inoculated plants were placed in a greenhouse for the development of powdery mildew at 25 and 20°C (day and night), relative humidity of 60%, and 16L/8D photoperiod. Control plants (mock-inoculated) were inoculated with the same volume of sterile water and maintained separately from the inoculated plants in the same greenhouse. The disease incidence, the disease index, the total lignin content, the total phenolic content, the hydrogen peroxide content, the lipid peroxidation content, the activity and gene expression level of phenylpropanoid pathway defense enzymes and ROS scavenging enzymes, and the expression level of SA signaling pathway marker genes at different time points were measured and calculated every 2 days after inoculation.

### Disease Incidence and Index Determination

The disease incidence and index of both Sixing F_1_ and Jin_12_ F_1_ cultivars were observed and recorded for both the inoculated and mock-inoculated plants every 2 days on 1, 3, 5, 7, 9, 11, and 13 days post inoculation (dpi). Five plants from each treatment and control were used as one independent replicate per time point. Twelve such replicates were set up per treatment. Disease severity was recorded on the individual pot of each cultivar. Based on the powdery mildew symptoms developed on the host plants, a scale of 1–9 of increasing disease severity was used according to the standard described by Liu et al. (2006).

Scale levels:

0: no symptoms;
1: the infected areas less than 30% in the front of the leaves and no symptoms in the reverse of the leaves;
3: the infected areas greater than 30% in the front of the leaves and less than 10% in the reverse of the leaves;
5: the infected areas greater than 30 and 10% in the front and reverse of the leaves, respectively, and a few lesions appeared on the petioles;
7: the powdery mildew covered in the front of the leaves and the infected areas greater than 10% in the reverse of the leaves, and more lesions appeared on the petioles and a few on the main stems;
9: the powdery mildew covered in the front of the leaves, petioles, and main stems, and the infected areas greater than 10% in the reverse of the leaves.

The calculation formulas for the disease incidence and index are as follows:

(1) Disease incidence (%)=(NIL/TNIL)×100

where NIL is the number of infected leaves, and TNIL is the total number of investigated leaves.

(2) Disease index=(Σ⁢NDL×GLDS)/(TNIL×THGL)×100

where NDL is the number of diseased leaves in each level; GLDS is the grade level of disease severity; TNIL is the total number of investigated leaves; and THGL is the highest-grade level.

### Leaf Cell Wall Isolation and Lignin Content Determination

The leaf cell walls were isolated according to the method described by Eskandari et al. (2018). Briefly, fresh leaf sample (0.5 g) was frozen and ground to powder in liquid nitrogen. The sample powder was homogenized in distilled water and then centrifuged at 10,000 g for 10 min. The precipitation was washed with absolute ethanol, rinsed with the mixture solution of chloroform and methanol (v/v = 1:2), and then washed with acetone three times. The cell wall pellet was filtered and finally dried overnight at 35°C. The residue (cell wall) was collected and kept at room temperature until use.

The content of lignin was determined and assayed by following the procedure of Iiyama and Wallis (1990). The cell wall preparation (5 mg) was treated at 70°C for 30 min with the mixture solution (2.5 ml) of 5% acetyl bromide and AcHO (w/w) and 0.1 ml of 70% HClO. After cooling, the reaction mixture was treated with 50 ml of 2 M NaOH and AcHO. The lignin content was determined by measuring the absorbance at 280 nm and calculated using a specific absorption coefficient of 20.0 g^–1^ l cm^–1^.

### Total Phenolics Content Determination

The content of total phenolics was measured according to the method described by Singleton and Rossi (1965) with a minor modification. The fresh leaf sample (0.5 g) was ground with quartz sand and then extracted with 70% ethanol (10 ml) for 10 min. The mixture was centrifuged at 12,000 g for 20 min. The absorbance of the supernatant at 760 nm was used to determine the total phenolic content and expressed as mg g^–1^ FW.

### Phenylpropanoid Pathway Enzyme Activity Determination

The fresh leaf sample (0.5 g) was homogenized in a 6 ml ice-cold borate buffer (5 mM, pH 8.8) using a pre-chilled mortar and pestle, and then centrifuged at 8,000 g for 20 min at 4°C. The supernatant was mixed with 0.02 M phenylalanine and distilled water and used as crude extracts. The extract was incubated at 30°C for 30 min and then measured at 290 nm for the determination of PAL activity (Ruiz et al., 1999; Hu et al., 2009), and at 420 nm for the determination of PPO activity following the oxidation of catechol (Chang et al., 2000). The activity of PAL and PPO was expressed as U min^–1^ g^–1^ FW.

### Hydrogen Peroxide (H_2_O_2_) and Lipid Peroxidation (MDA) Content Determination

For the determination of the H_2_O_2_ content in the leaves of different pumpkin cultivars, the fresh leaf sample (0.5 g) was homogenized in 5 ml of precooled HClO_4_ (1.0 M) using the pre-chilled mortar and pestle and then centrifuged at 10,000 g for 10 min. The content of H_2_O_2_ was determined and calculated according to the method described by Willekens et al. (1997) and expressed as μmol g^–1^ FW.

The level of lipid peroxidation was determined by quantifying the MDA accumulation in the leaves of different pumpkin cultivars according to the method described by Hodges et al. (1999) and Tian et al. (2015) with some modifications. Briefly, the fresh leaf sample (0.5 g) was homogenized in 2.5 ml of 0.1% trichloroacetic acid and then centrifuged at 10,000 g for 15 min. The absorbance of the supernatant was recorded at a 532-nm wavelength. The content of MDA was expressed as nmol g^–1^ FW.

### ROS Scavenging Enzyme Activity Determination

The fresh leaf sample (1 g) was homogenized in an ice-cold buffer (pH 7.8) with 10 ml of 25 mM potassium phosphate containing 0.2 mM EDTA and 2% polyvinylpyrrolidone. The homogenate was centrifuged at 10, 000 g for 25 min at 4°C, and then the supernatant was used as an enzyme extract to determine the activity of ROS scavenging enzymes (SOD, POD, CAT, and APX). All spectrophotometric analyses were conducted on a spectrophotometer (SP-756P, Shanghai, China). The activity of ROS scavenging enzymes was expressed as U g^–1^ min^–1^ FW.

The SOD activity was measured according to the method of Giannopolitis and Ries (1977) with a minor modification. The POD activity was determined and assayed according to the method described by Chance and Maehly (1955) and Cakmak and Marschner (1992) with a minor modification. The CAT activity was measured according to Patra et al. (1978) by measuring the decrease in the amount of the H_2_O_2_ decomposing at the absorbance of 240 nm. The APX activity was measured according to Nakano and Asada (1981) by estimating the rate of ascorbate oxidation at 290 nm.

### Total RNA Extraction and First-Strand cDNA Synthesis

The leaf samples were collected every 2 days post inoculation (dpi) for both inoculated and mock-inoculated seedlings on 1, 3, 5, 7, 9, 11, and 13 days dpi from both Sixing F_1_ and Jin_12_ F_1_ cultivars for total RNA extractions. Five plants from each treatment were used as one independent replicate per time point. Total RNA extraction and first-strand cDNA synthesis were carried out according to the methods described by Zhang et al. (2016).

### Real-Time Quantitative PCR (RT-qPCR) Analysis

The gene expression level of phenylpropanoid pathway defense enzymes (*PAL* and *PPO*) (Bezold et al., 2005; Zhu et al., 2018), ROS scavenging enzymes (*SOD*, *POD*, *CAT*, *APX*) (Yao et al., 2018; Liu et al., 2020), and SA signaling pathway marker genes (*PR1, PR2* and *ICS1*) (Wang et al., 2010; Yan, 2018; Luan et al., 2019) was determined in pumpkin leaves inoculated with *P. xanthii* and mock-inoculated pumpkin leaves at different time points after inoculation. The procedure of RT-qPCR was performed following the methods of Zhang et al. (2016). The sequences of the primers used in the RT-qPCR analyses were designed using the Primer Express 3.0 software based on the sequences of target genes in NCBI and listed in Table 1. The actin gene of pumpkin was used as an internal control (Bezold et al., 2005). The gene expression level was determined using the method of 2^–ΔΔCt^ (Livak and Schmittgen, 2001).

**TABLE 1:** Gene-specific PCR primers for target genes and Actin gene.

**Genes name**	**NCBI accession number**	**Primers sequence**	**References**
PAL (F)	CD726828.1	5′-AACTTCTCCTCAATGGCTTGGT-3′	Bezold et al., 2005
PAL (R)		5′-TGAAACATCAATCAAAGGGTTG-3′	
PPO (F)	KR819890	5′-CTAGCCGTGGAAACCGA	Zhu et al., 2018
PPO (R)		5′-TGATTGGCTCACAGTGGA	
POD (F)	MF988305	5′-TTTTATTTGGAGCCTCTTATGC-3′	Liu et al., 2020
POD (R)		5′-GTCCGTTGAGTTCACTGTCG-3′	
SOD (F)	AF009734	5′-GTCTACTGGACCACATTACAACC-3′	Yao et al., 2018
SOD (R)		5′-CACAACAGCCCTTCCGATAA-3′	
CAT (F)	D55646	5′-CGCAAGAAGATCGTGTTCAA-3′	Yao et al., 2018
CAT (R)		5′-CTTAGGAAGCAACAAAGGCG-3′	
APX (F)	KF954415	5′-GCCTTGACATTGCTGTTA-3′	Yao et al., 2018
APX (R)		5′-GAACCCTTGGTAGCATCA-3′	
PR1 (F)	DQ641122	F: AACTCTGGCGGACCTTAC	Wang et al., 2010
PR1 (R)		R: GACTTCCTCCACACTACT	
PR2 (F)	XM_008445504.2	F: TCTTGGTCTTCTTGTGCC	Luan et al., 2019
PR2 (R)		R: GAGCATCAAGTGAACCTC	
ICS1 (F)	–	F: GCATTCACCTCCGGGATTAT	Yan, 2018
ICS1 (R)		R: AAGGTGCGAGGAAGATG	
ACT (F)	–	5′-TgYgACAATggAACWggAATg-3′	Bezold et al., 2005
ACT (R)		5′-CATCTgYTggAARgTgCTgAg-3′	

*F represents forward; R represents reverse.*

### Statistical Analysis

The data were subjected to variance analysis (ANOVA) using SPSS Version 16.0 (SPSS Inc., Chicago, IL). Each treatment had 12 replications. Duncan’s multiple range test was computed using the standard error and T values of adjusted degrees of freedom. The differences between treatments were considered significant at the level of *P* < *0.05*.

## Results

### Symptoms of *C. pepo* After Inoculation With *P. xanthii*

**FIGURE 1:**
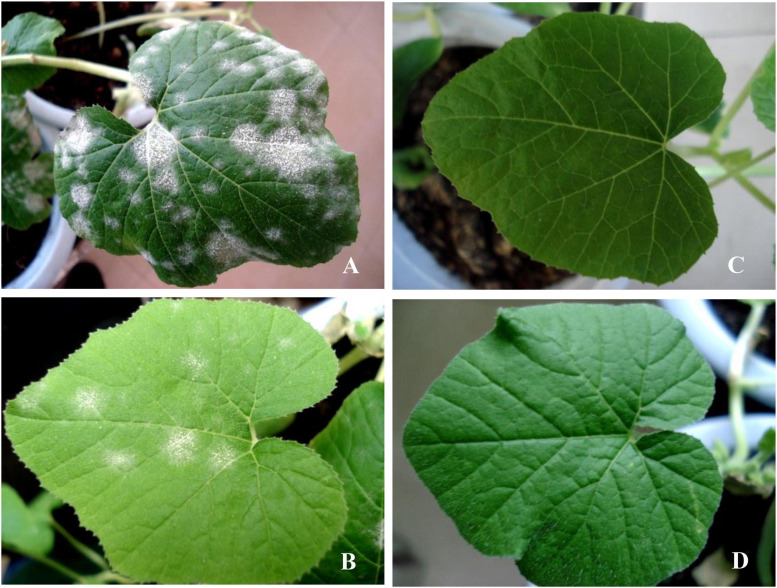
The symptoms of different cultivars of *Cucurbita pepo* on the 13^th^ day after inoculation with the pathogen of *Podosphaera xanthii*. **(A)** The cultivar of Jin_12_ F_1_ after inoculation with *P. xanthii*; **(B)** the cultivar of Sixing F_1_ after inoculation with *P. xanthii*; **(C)** the cultivar of Jin_12_ F_1_ after inoculation with sterile water but not *P. xanthii*; and **(D)** the cultivar of Sixing F_1_ after inoculation with sterile water but not *P. xanthii*.

A large number of diseased powdery areas and a layer of white powdery mildew were observed on the surface of Jin_12_ F_1_ leaves (Figure 1A) on the 13^th^ day after inoculation with *P. xanthii* in comparison to the leaves of the mock-inoculated Jin_12_ F_1_ seedlings (Figure 1C). However, small and sparse powdery areas were observed on the leaves of the Sixing F_1_ cultivar (Figure 1B), and no diseased powdery area was observed on the leaves of the mock-inoculated Sixing F_1_ (Figure 1D).

### Disease Severity of *C. pepo* After Inoculation With *P. xanthii*

The cultivars of Jin_12_ F_1_ and Sixing F_1_ inoculated with *P. xanthii* begun to show the disease symptoms at the fifth and seventh day after the inoculation. The disease incidence and index were significantly different between the cultivars of Sixing F_1_ and Jin_12_ F_1_ (Tables 2, 3) (*P* < 0.05) after inoculation with *P. xanthii*. In contrast, the mock-inoculated seedlings grew normally and had no disease symptoms during the inoculation.

**TABLE 2:** The disease incidence of different cultivars of Cucurbita pepo after inoculation with *Podosphaera xanthii*.

**Cultivars**	**Treatments**	**Days post inoculation (dpi)**						
		**1**	**3**	**5**	**7**	**9**	**11**	**13**
		**Disease incidence (%)**						
Sixing F1	Treatment	0.0 a	0.0 a	0.0 b	6.7 b	16.7 b	21.3 b	22.3 b
	Control	0.0 a	0.0 a	0.0 b	0.0 c	0.0 c	0.0 c	0.0 c
Jin12 F1	Treatment	0.0 a	0.0 a	3.3 a	26.7 a	60.0 a	76.7 a	80.0 a
	Control	0.0 a	0.0 a	0.0 b	0.0 c	0.0 c	0.0 c	0.0 c

*Data are means of 12 replicates. Different letters in the same column denote significant differences at the P < 0.05 level by Duncan’s new multiple range test (n = 12). In the two treatments, pumpkin seedling leaves were inoculated with P. xanthii, whereas in the two controls, pumpkin seedling leaves were inoculated with sterile water but not P. xanthii.*

**TABLE 3:** The disease index of different cultivars of *Cucurbita pepo* after inoculation with *Podosphaera xanthii.*

**Cultivars**	**Treatments**	**Days post inoculation (dpi)**						
		**1**	**3**	**5**	**7**	**9**	**11**	**13**
		**Disease index**						
Sixing F_1_	Treatment	0.0 a	0.0 a	0.0 b	4.7 b	13.3 b	16.4 b	17.7 b
	Control	0.0 a	0.0 a	0.0 b	0.0 c	0.0 c	0.0 c	0.0 c
Jin_12_ F_1_	Treatment	0.0 a	0.0 a	3.3 a	16.7 a	40.7 a	46.0 a	72.6 a
	Control	0.0 a	0.0 a	0.0 b	0.0 c	0.0 c	0.0 c	0.0 c

*Data are means of 12 replicates. The different letters and treatments are detailed in the footnote of Table 2.*

The disease incidence and index of two pumpkin cultivars were significantly and continuously increased by the increase in the inoculation time (7, 9, 11, and 13 days). The disease incidence and index of Jin_12_ F_1_ cultivar were significantly higher than those of the Sixing F_1_ cultivar. At day 13, the disease incidence and index of the Jin_12_ F_1_ cultivar were 80.0% and 72.6, respectively, whereas they were only 22.3% and 17.7 in the Sixing F_1_ cultivar, respectively. In addition, the Jin_12_ F_1_ cultivar begun to show symptoms at the fifth day after inoculation, while the Sixing F_1_ cultivar begun to show symptoms at the seventh day after inoculation. Furthermore, the expansion speed of the diseased powdery area in the Jin_12_ F_1_ cultivar was faster than in the Sixing F_1_ cultivar with the increase in the inoculation time (Tables 2, 3).

### Lignin and Total Phenolics Contents in Pumpkin Seedlings

The lignin and total phenolics contents in the leaves of the cultivars of Sixing F_1_ and Jin_12_ F_1_ were increased from 1 to 9 or 11 days by the treatment with *P. xanthii* and peaked on the 9^th^ and 11^th^ days. The levels of lignin and total phenolics of the Sixing F_1_ cultivar were significantly higher in comparison to those of the Jin_12_ F_1_ cultivar. The average contents of lignin and total phenolics on the 9^th^ to 11^th^ days were significantly increased by the inoculation to 21.24 and 21.09% in the leaves of the Sixing F_1_ cultivar and to 12.38 and 18.65% in the leaves of the Jin_12_ F_1_ cultivar in comparison to the control, respectively (Table 4).

**TABLE 4:** Lignin and total phenolic content in different cultivars of *Cucurbita pepo* seedlings after inoculation with *Podosphaera xanthii.*

**Cultivars**	**Treatments**	**Days post inoculation (dpi)**						
		**1**	**3**	**5**	**7**	**9**	**11**	**13**
		**Lignin content (% of cell wall dry weight)**						
Sixing F_1_	Treatment	2.56 a	2.89 a	3.28 a	5.08 a	6.56 a	6.52 a	6.13 a
	Control	2.43 b	2.78 b	3.09 b	4.25 b	5.34 b	5.45 b	5.25 b
Jin_12_ F_1_	Treatment	2.12 c	2.24 c	2.67 c	3.11 c	3.98 c	4.02 c	3.96 c
	Control	2.01 c	2.15 c	2.48 d	2.81 d	3.51 d	3.61 d	3.74 d
		**Total phenolics content (mg g^−1^ FW)**						
Sixing F_1_	Treatment	3.62 a	3.85 a	3.98 a	4.29 a	4.79 a	4.81 a	4.25 a
	Control	3.35 b	3.42 b	3.62 b	3.98 b	4.02 b	3.91 c	4.06 b
Jin_12_ F_1_	Treatment	3.34 b	3.57 b	3.72 b	3.89 b	4.21 b	4.33 b	3.98 bc
	Control	3.01 c	3.12 c	3.23 c	3.45 c	3.64 c	3.56 d	3.54 c

*Data are means of 12 replicates. The different letters and treatments are detailed in the footnote of Table 2.*

### Activity of the Phenylpropanoids Pathway Defense Enzymes

**FIGURE 2:**
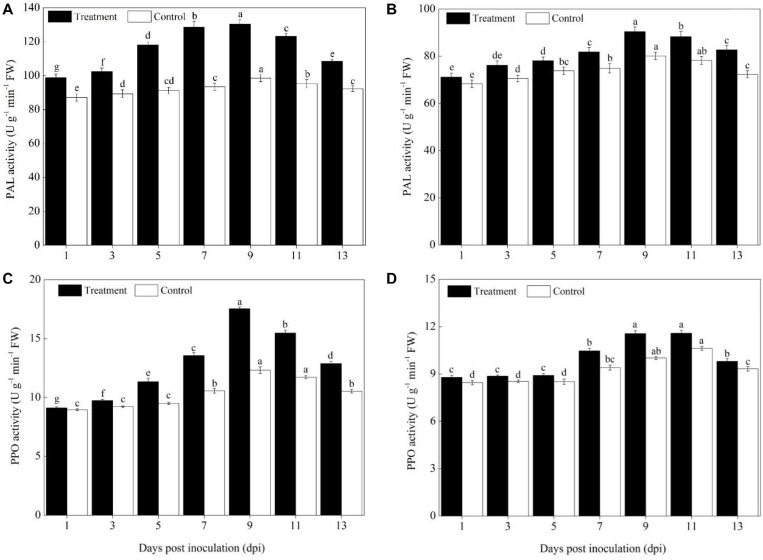
PAL and PPO activity in the leaves of Sixing F_1_ and Jin_12_ F_1_ at different time points after inoculation with *Podosphaera xanthii.* **(A)** The PAL activity in the leaves of Sixing F_1_; **(B)** the PAL activity in the leaves of Jin_12_ F_1_; **(C)** the PPO activity in the leaves of Sixing F_1_; and **(D)** the PPO activity in the leaves of Jin_12_ F_1_. The line bars represent the standard errors of the means. Different letters denote significant difference at the *P* < *0.05* level by Duncan’s new multiple range test (*n* = 12). The treatments are detailed in the footnote of Table 2.

The activities of the phenylpropanoids pathway enzymes PAL and PPO in different pumpkin cultivars (Sixing F_1_ and Jin_12_ F_1_) were increased significantly on the 5th day after the *P. xanthii* inoculation, peaked on the 9^th^ or 11^th^ day, and then declined gradually (Figure 2). However, the activity of PAL and PPO differed significantly between the Sixing F_1_ cultivar and the Jin_12_ F_1_ cultivar. A higher level of PAL and PPO activity was detected in Sixing F_1_ leaves than in Jin_12_ F_1_ leaves. Compared with those of the untreated control, the PAL and PPO activities in the leaves of the Sixing F_1_ cultivar were increased by 32.52% (Figure 2A) and 42.42% (Figure 2C) on the 9^th^ day, respectively. In contrast, the PAL and PPO activities in the leaves of the Jin_12_ F_1_ cultivar were increased by 12.84% (Figure 2B) and 15.43% (Figure 2D) on the 9^th^ day, respectively.

### Hydrogen Peroxide (H_2_O_2_) and Lipid Peroxidation (MDA) Contents in Pumpkin Seedling

The H_2_O_2_ and MDA contents of Sixing F_1_ and Jin_12_ F_1_ seedling leaves were increased with the duration of post inoculation with *P. xanthii*, peaked on the 9^th^ and 11^th^ days, and then declined gradually. The H_2_O_2_ and MDA contents in the Jin_12_ F_1_ leaves were significantly higher than those in the Sixing F_1_ leaves. The maximum H_2_O_2_ and MDA contents were increased significantly by 26.83 and 26.42% in the Sixing F_1_ leaves and 27.08 and 28.32% in the Jin_12_ F_1_ leaves on the 9^th^ and 11^th^ days after inoculation with *P. xanthii*, respectively, compared with those of control leaves inoculated with sterile water (Table 5).

**TABLE 5:** H_2_O_2_ and MDA content in different cultivars of *Cucurbita pepo* seedlings after inoculation with *Podosphaera xanthii.*

**Cultivars**	**Treatments**	**Days post inoculation (dpi)**						
		**1**	**3**	**5**	**7**	**9**	**11**	**13**
		**H_2_O_2_ (μ mol g^–1^ FW)**						
Sixing F_1_	Treatment	0.25 c	0.28 b	0.39 a	0.46 b	0.52 b	0.47 b	0.38 c
	Control	0.23 d	0.25 c	0.32 c	0.37 d	0.41 d	0.43 c	0.35 d
Jin_12_ F_1_	Treatment	0.29 a	0.31 a	0.37 b	0.48 a	0.61 a	0.58 a	0.49 a
	Control	0.27 b	0.28 b	0.32 c	0.41 c	0.48 c	0.48 b	0.44 b
		**MDA (nmol g^–1^ FW)**						
Sixing F_1_	Treatment	2.45 c	2.91 b	3.45 b	4.05 c	4.61 c	4.69 c	4.35 c
	Control	2.21 d	2.45 c	2.88 c	3.27 d	3.68 d	3.71 d	3.57 d
Jin_12_ F_1_	Treatment	2.87 a	3.35 a	3.68 a	5.07 a	5.31 a	6.66 a	5.98 a
	Control	2.64 b	2.85 b	3.56 b	4.68 b	5.08 b	5.19 b	4.94 b

*Data are means of 12 replicates. The different letters and treatments are detailed in the footnote of Table 2.*

### Activity of ROS Scavenging Defense Enzymes

**FIGURE 3:**
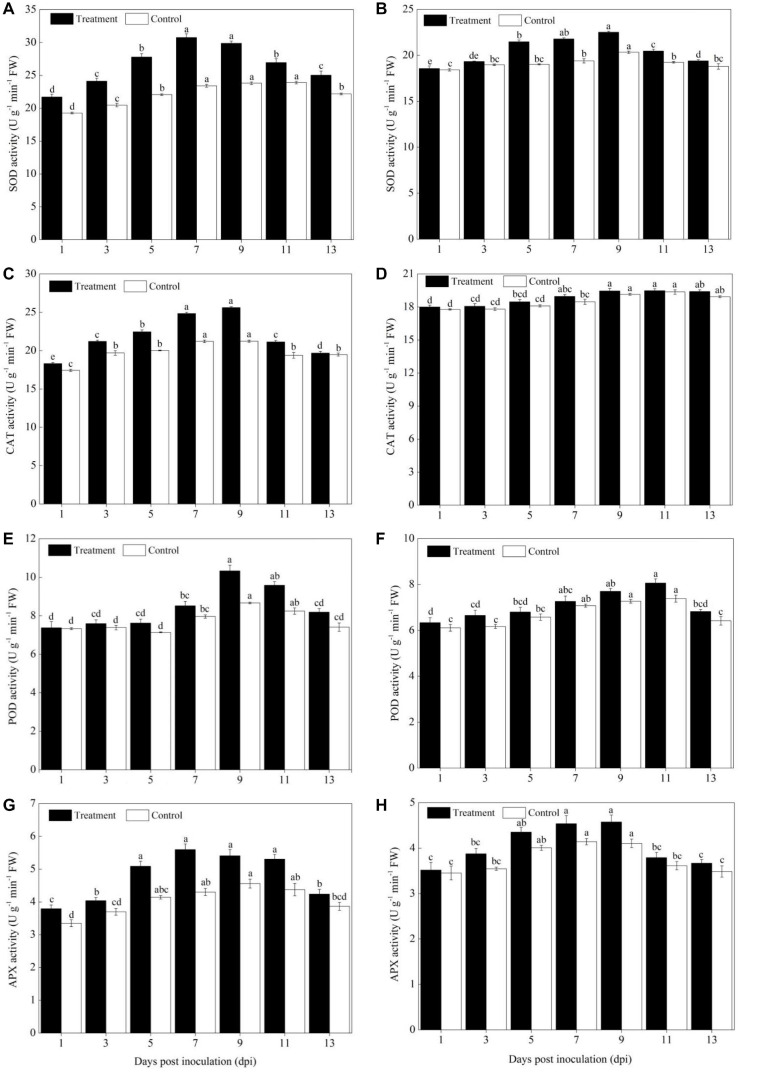
Activities of ROS scavenging enzymes in the leaves of Sixing F_1_ and Jin_12_ F_1_ at different time points after inoculation with *Podosphaera xanthii.* **(A,C,E,G)** The activity of SOD, CAT, POD, and APX in Sixing F_1_, respectively; **(B,D,F,H)** the activity of SOD, CAT, POD, and APX in Jin_12_ F_1_, respectively. The line bars, different letters, and the treatments are detailed in the footnote of Figure 2 and Table 2.

The activity of ROS scavenging enzymes (SOD, POD, CAT, and APX) was significantly increased in the cultivars of Sixing F_1_ and Jin_12_ F_1_ after being inoculated with the pathogen of *P. xanthii* from the 3^rd^ to 11^th^ day in comparison to the control (Figure 3). In addition, a higher activity of ROS scavenging enzymes was observed in the leaves of the Sixing F_1_ cultivar in comparison to the Jin_12_ F_1_ cultivar.

The SOD induction reached its maximum from the 7^th^ to 9^th^ day in the leaves of Sixing F_1_ and Jin_12_ F_1_ after inoculation and then declined. Compared with the control seedlings inoculated with sterile water, the average SOD activity was increased by 28.47% in the Sixing F_1_ leaves (Figure 3A) and 11.48% in the Jin_12_ F_1_ leaves (Figure 3B) on the 7^th^ and 9^th^ days after inoculation with *P. xanthii*. In addition, the activity of SOD in the leaves of the Sixing F_1_ cultivar was significantly higher than in the leaves of the Jin_12_ F_1_ cultivar. The average SOD activity in the Sixing F_1_ leaves was 30.33 U g^–1^ min^–1^ FW, whereas it was 22.15 U g^–1^ min^–1^ FW in the Jin_12_ F_1_ leaves on the 7^th^ and 9^th^ days after inoculation.

The CAT activity in the Sixing F_1_ leaves was significantly increased up to the 7^th^ and 9^th^ days, whereas in the Jin_12_ F_1_ leaves, it was significantly increased up to the 9^th^ and 11^th^ days after inoculation, and then declined in all the treatments. However, the CAT activity of the Sixing F_1_ cultivar was significantly higher than that of the Jin_12_ F_1_ cultivar. The CAT activity on the 9^th^ day after inoculation was 25.61 U g^–1^ min^–1^ FW in the Sixing F_1_ leaves, whereas it was 19.45 U g^–1^ min^–1^ FW in the Jin_12_ F_1_ leaves. The CAT activity on the 9^th^ day after being inoculated with *P. xanthii* was increased by 20.65% in Sixing F_1_ leaves (Figure 3C), whereas it was increased by 1.57% in the Jin_12_ F_1_ leaves (Figure 3D) in comparison to those in the control leaves inoculated with sterile water but not *P. xanthii*.

The inoculation of *P. xanthii* induced the maximum (peak) level of POD activity in the cultivars of Sixing F_1_ and Jin_12_ F_1_ on the 9^th^ and 11^th^ days, respectively, and thereafter it declined. The POD activity in the Sixing F_1_ leaves was increased by 19.19% (Figure 3E) on the 9^th^ day and 9.21% in the Jin_12_ F_1_ leaves (Figure 3F) on the 11^th^ day after being inoculated with *P. xanthii* in comparison to the control seedlings inoculated with sterile water. It was significantly higher in the Sixing F_1_ cultivar than in the Jin_12_ F_1_ cultivar, with a maximum activity of 10.33 U g^–1^ min^–1^ FW in the Sixing F_1_ leaves on the 9^th^ day and of 8.07 U g^–1^ min^–1^ FW in the Jin_12_ F_1_ leaves on the 11^th^ day after inoculation.

Increased activities of APX were observed on the leaves of the Sixing F_1_ and Jin_12_ F_1_ cultivars by the inoculation with *P. xanthii*, and the induction reached its maximum on the 7^th^ and 9^th^ days and thereafter it declined. The APX activity was 5.60 U g^–1^ min^–1^ FW and 5.41 U g^–1^ min^–1^ FW in the Sixing F_1_ leaves and 4.54 U g^–1^ min^–1^ FW and 4.57 U g^–1^ min^–1^ FW in the Jin_12_ F_1_ leaves on the 7^th^ and 9^th^ days after inoculation, respectively. The APX activity was increased by 30.05 and 18.64% in the Sixing F_1_ leaves (Figure 3G) and by 9.57 and 11.44% in the Jin_12_ F_1_ leaves (Figure 3H) on the 7^th^ and 9^th^ days, respectively, after being inoculated with *P. xanthii* in comparison to the control seedlings inoculated with sterile water.

### Levels of Defense Genes Expression

**FIGURE 4:**
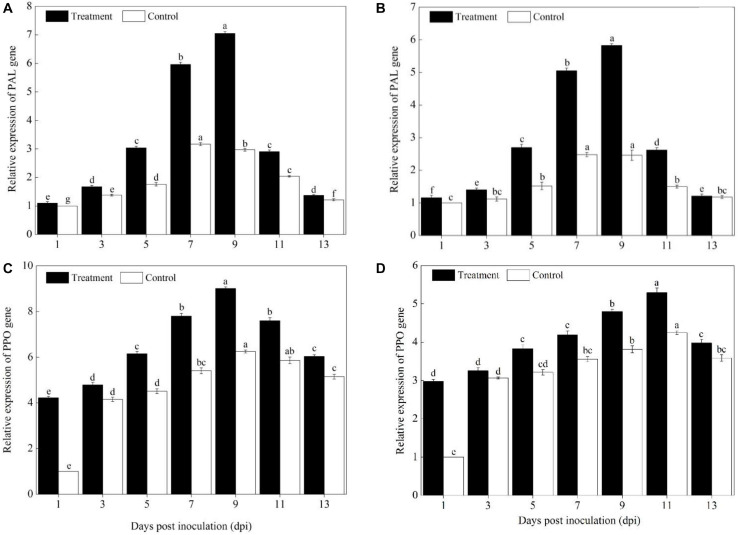
Expression levels of *PAL* and *PPO* genes in the leaves of Sixing F_1_ and Jin_12_ F_1_ at different time points after being inoculated with *Podosphaera xanthii.* **(A,B)** The expression levels of the *PAL* gene in Sixing F_1_ and Jin_12_ F_1_, respectively; **(C,D)** the expression levels of the *PPO* gene in Sixing F_1_ and Jin_12_ F_1_, respectively. The line bars, different letters, and treatments are detailed in the footnote of Figure 2 and Table 2.

**FIGURE 5:**
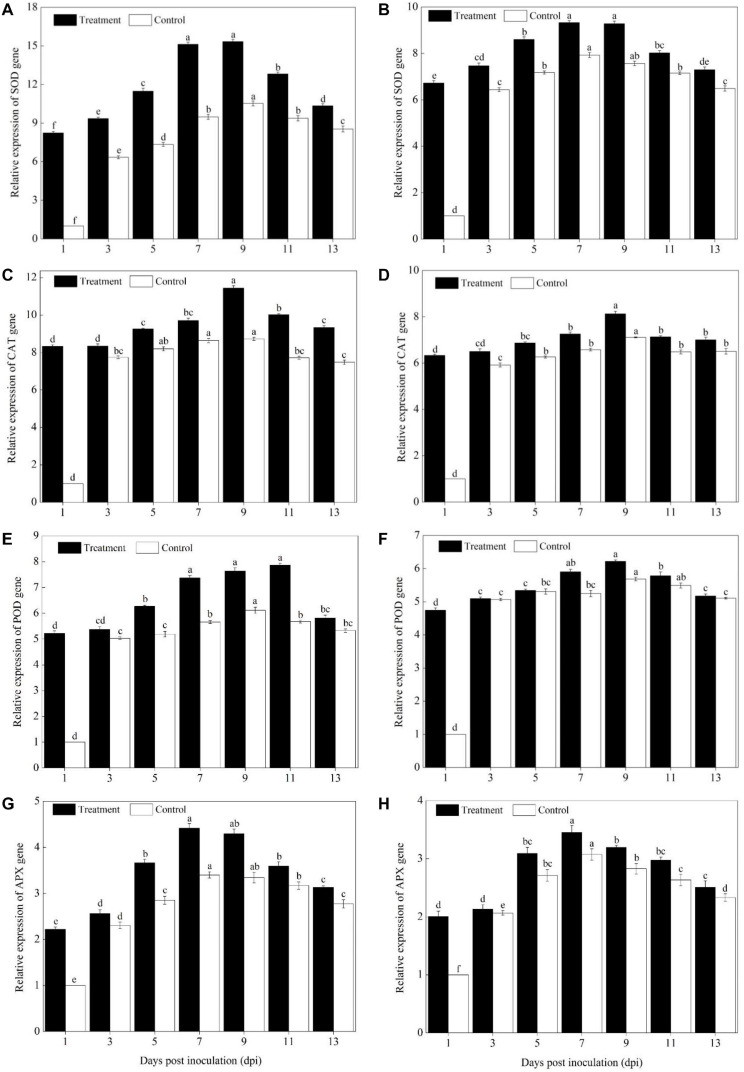
Expression levels of ROS scavenging enzyme genes in the leaves of Sixing F_1_ and Jin_12_ F_1_ at different time points after being inoculated with *Podosphaera xanthii.* **(A,C,E,G)** The expression levels of *SOD*, *CAT*, *POD*, and *APX* genes in Sixing F_1_, respectively; **(B,D,F,H)** the expression levels of *SOD*, *CAT*, *POD*, and *APX* genes in Jin_12_ F_1_, respectively. The line bars, different letters, and treatments are detailed in the footnote of Figure 2 and Table 2.

**FIGURE 6:**
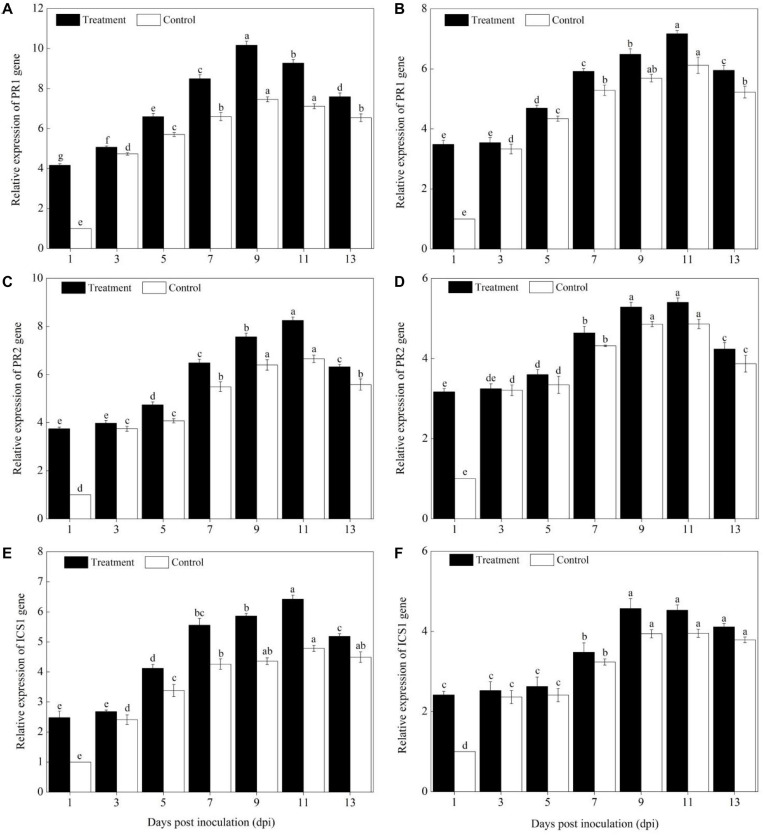
Expression levels of SA signaling pathway marker genes in the leaves of Sixing F_1_ and Jin_12_ F_1_ at different time points after being inoculated with *Podosphaera xanthii.* **(A,C,E)** The expression levels of *PR1, PR2*, and *ICS1* genes in Sixing F_1_, respectively; **(B,D,F)** the expression levels of *PR1, PR2*, and *ICS1* genes in Jin_12_ F_1_, respectively. The line bars, different letters, and treatments are detailed in the footnote of Figure 2 and Table 2.

Compared with pumpkin leaves inoculated with sterile water, the expression levels of *PAL, PPO, SOD, POD, CAT, APX, PR1, PR2*, and *ICS1* genes in the Sixing F_1_ and Jin_12_ F_1_ leaves were significantly upregulated after inoculation with *P. xanthii* at different time points (Figures 4–6). The expression levels of *PAL, PPO, SOD, CAT, POD, APX, PR1, PR2*, and *ICS1* genes reached their maximum on the 7^th^, 9^th^, and 11^th^ days after inoculation, and thereafter, they declined gradually in all the treatments. Also, there were significant differences in the expression levels of *PAL, PPO, SOD, POD, CAT, APX, PR1, PR2*, and *ICS1* genes between the Sixing F_1_ cultivar and the Jin_12_ F_1_ cultivar at different time points after inoculation. The expression levels of *PAL* (Figure 4A), *PPO* (Figure 4C), *SOD* (Figure 5A), *CAT* (Figure 5C), *POD* (Figure 5E), *APX* (Figure 5G), *PR1* (Figure 6A), *PR2* (Figure 6C), and *ICS1* (Figure 6E) genes in the Sixing F_1_ leaves were significantly higher than those in the Jin_12_ F_1_ leaves (Figures 4B,D, 5B,D,F,H, 6B,D,F). The average expression levels of *PAL, PPO, SOD, CAT, POD, APX, PR1, PR2*, and *ICS1* genes in the Sixing F_1_ leaves were 1. 16-, 1. 61-, 1. 46-, 1. 35-, 1. 19-, 1. 23-, 1. 38-, 1. 39-, and 1.33-fold higher than those in the Jin_12_ F_1_ leaves, respectively, at each sampling time from 1 to 13 days after inoculation.

## Discussion

Previous studies demonstrated that disease resistance screening was important to get tolerant germplasm for breeding new cultivars to control powdery mildew in field and greenhouse-grown pumpkins (Luitel et al., 2016; Rabelo et al., 2017). Furthermore, the activation speeds and activity levels of defense enzymes vary in different plant genotypes or plant–pathogen interactions (Yan et al., 2009). Therefore, the changes in the levels of these enzymes could serve as valuable physiological indices for the selection of tolerant germplasms. In the present study, we evaluated the ability of two commercial pumpkin cultivars in resistance to *P. xanthii* infection. Our results demonstrate that the Sixing F_1_ cultivar has a much higher ability in resistance to *P. xanthii* infection than the Jin_12_ F_1_ cultivar and can be considered as a tolerant variety. In addition, our results showed that the disease incidence and index were significantly higher than the Sixing F_1_ cultivar at different time points after inoculation. The Jin_12_ F_1_ cultivar is a susceptible variety because of its high disease incidence and index.

Our comparative study of tolerant and susceptible cultivars provides a new insight for the mechanisms of different pumpkin cultivars in resistance to *P. xanthii* infection. The Sixing F_1_ cultivar had less ROS accumulation, lower rates of lipid peroxidation, a higher level of lignin and total phenolics contents, higher PAL, PPO activity, and genes expression, and a higher expression of SA signaling pathway marker genes than the Jin_12_ F_1_ cultivar. In addition, a positive relationship was discovered between the high H_2_O_2_ and MDA contents and the disease incidence and index. Thus, the possible mechanism for the stronger resistance of the Sixing F_1_ cultivar to *P. xanthii* infection may be through enhancing the activity and gene expression of the phenylpropanoids pathway and ROS scavenging and the SA signaling pathway by increasing phenolic substances and lignin contents and reducing ROS accumulation. To the best of our knowledge, this is the first time that it was suggested that the pumpkin cultivars are resistant to *P. xanthii* infection through stimulating the phenylpropanoids and the SA pathway and antioxidative defense systems. These are also supported by previous studies.

Phenylpropanoid pathway defense enzymes (PAL, POX, and PPO) are known to be involved in plant disease resistance (Paparu et al., 2010; Babu et al., 2015; Jia et al., 2016) and closely related to plant resistance to *P. xanthii* of cucumber (Chen et al., 2014; Gao et al., 2019) and plant-induced systemic resistance (Mauch-mani and Slusarenko, 1996). Among all the phenylpropanoid pathway defense enzymes, PAL is the first enzyme that produces the precursors for lignin and phenolic secondary metabolites (Sticher et al., 1997). PPO is another key enzyme in the synthesis of phenolic compounds with antimicrobial activity (Babu et al., 2015; Jia et al., 2016; Niu et al., 2018). Muslim et al. (2019) revealed that the lignin deposition in cucumber plants can prevent the pathogen *Colletotrichum orbiculare* infection, and also the level of total phenolics was increased in cucumber seedlings after being inoculated with the *P. xanthii* (Chen et al., 2014).

The ROS-scavenging defense enzymes play an essential role in adjusting the extent of oxidative damage (Gao et al., 2020). Our present study found that the expression levels of ROS scavenging defense enzymes and genes (*SOD*, *POD*, *CAT*, and *APX*) were significantly increased in the tolerant cultivar of Sixing F_1_ after *P. xanthii* inoculation in agreement with the results of Wang et al. (2010) in the powdery mildew pathogen infested cucumber. H_2_O_2_ and MDA have been considered as the important types of ROS and key biochemical indicators of oxidative damage in plants against pathogen infection (Garcia-Limones et al., 2002; Mellersh et al., 2002; Gill and Tuteja, 2010; Mandal et al., 2011; Chavan et al., 2013). El-Komy (2014) demonstrated that the resistant cultivar faba bean (*Vicia faba*) showed less ROS accumulation, a lower rate of lipid peroxidation, and higher activity of the enzymatic ROS scavenging system compared with the susceptible cultivar during the interaction of *Botrytis fabae*. The ability of tomato (*Lycopersicon esculentum* L.) in resistance to *Botrytis cinerea* infection resulted from the early induction of H_2_O_2_ (Patykowski and Urbanek, 2003). Meanwhile, the excess H_2_O_2_ can lead to the peroxidation of unsaturated lipids of membranes in plants (Apel and Hirt, 2004) and the increased contents of MDA in both the resistant and susceptible cultivars of the faba bean during the interaction between *B. fabae* and faba bean (El-Komy, 2014).

Previous studies demonstrated that plant hormones of SA act as an important signal molecule in plant resistance to pathogen infection (Vlot et al., 2009). Meanwhile, similar studies revealed that the isochorismate synthase (*ICS1/SID2*) gene plays the main role in SA accumulation, and *PR* gene acted as reliable markers of SA-mediated response or SAR as well as basal content maintenance under the normal condition (Hunt and Ryals, 1996; Garcion et al., 2008; Dempsey et al., 2011). Li et al. (2019) discovered that the basal defense and SA-signaling-associated pathways play an important role in contributing to the postpenetration defense against tobacco powdery mildew (*Golovinomyces cichoracearum*) in *Arabidopsis* by comparative transcriptome analysis. Wu et al. (2019) found that *NPR1* and *ICS1/SID2* genes can activate the SA pathway of *Arabidopsis thaliana*, and the increased expression of SA pathway marker genes *PR2* and *PR5* in *A. thaliana* plays a positive role in defense against (hemi)-biotrophs after the cold treatment.

## Conclusion

Our study suggests that the Sixing F_1_ cultivar can be considered as a tolerant cultivar, and the Jin_12_ F_1_ cultivar can be considered as a susceptible cultivar. One possible mechanism to activate the defense system and to prevent the pathogen infection of the pumpkin cultivars in resistance to *P. xanthii* infection is through enhancing the activity and gene expression of the enzymes involved in the phenylpropanoids pathway to promote the synthesis of phenolic substances and lignin and in ROS scavenging to reduce the ROS accumulation and to balance the oxidative damage, and in the SA signaling pathway to activate plant resistance to pathogen infection. However, more research is needed to determine other defense genes in pumpkins that encode the pathogenesis-related proteins and plant hormones in resistance to *P. xanthii* infection in the future.

## Data Availability Statement

All datasets generated for this study are included in the article/supplementary material, further inquiries can be directed to the corresponding author/s.

## Author Contributions

SZ conceived the experiments with the help of BX. SZ collected and prepared the fungus and pumpkin seedling samples, performed *Podosphaera xanthii* infections, extracted the total RNAs, and wrote the manuscript. JL and SZ performed RT-qPCR and analyzed the data. SZ and JZ interpreted the results. JZ made critical revising, editing, and proofreading. SZ and BX revised and approved the final manuscript. All authors contributed to the article and approved the submitted version.

## Conflict of Interest

The authors declare that the research was conducted in the absence of any commercial or financial relationships that could be construed as a potential conflict of interest.
